# Association of Individual and Familial History of Correctional Control With Health Outcomes of Patients in a Primary Care Center

**DOI:** 10.1001/jamanetworkopen.2021.33384

**Published:** 2021-11-08

**Authors:** Onagh MacKenzie, Jacqueline Goldman, Madeline Chin, Bridget Duffy, Sarah Martino, Susan Ramsey, Monik C. Jiménez, Rahul Vanjani

**Affiliations:** 1 Division of General Internal Medicine, Warren Alpert Medical School of Brown University, Providence, Rhode Island; 2 Department of Epidemiology, Brown University School of Public Health, Providence, Rhode Island; 3 Center for Health and Justice Transformation, The Miriam Hospital, Providence, Rhode Island; 4 Division of General Internal Medicine, Rhode Island Hospital, Providence; 5 Division of Women’s Health, Brigham and Women’s Hospital, Boston, Massachusetts

## Abstract

**Importance:**

Research has shown that experiences of incarceration, probation, and parole are associated with worse health outcomes for incarcerated individuals and their families.

**Objectives:**

To quantify the proportion of patients in an urban primary care clinic with an individual or family history of incarceration, probation, and/or parole and to evaluate how correctional control is associated with subjective and objective health outcomes.

**Design, Setting, and Participants:**

This cross-sectional, mixed-methods study used patient surveys and retrospective medical record review to assess the experience of correctional control among 200 English-speaking adult patients presenting for care at the Rhode Island Hospital Center for Primary Care between July 9, 2019, and January 10, 2020.

**Main Outcomes and Measures:**

Patient surveys included closed and open-ended questions pertaining to personal or familial experiences of incarceration, probation, and parole, as well as health outcomes associated with these experiences. Medical record review abstracted key health indicators and health care use data.

**Results:**

In this cross-sectional study of 200 adult patients (1 participant was removed from the full analytic sample owing to missing ethnicity data; 113 of 199 men [56.8%]; mean [SD] age, 51.2 [14.0] years) presenting for primary care, 78 of 199 (39.2%) had a history of incarceration, 32 of 199 (16.1%) were on probation or parole at the time of the study, and 92 of 199 (46.2%) reported having a family member with a history of incarceration. Of the 199 patients, 62 (31.2%) identified as non-Hispanic Black, 93 (46.7%) identified as non-Hispanic White, and 44 (22.1%) identified as belonging to another race (American Indian and Alaska Native, Asian, Native Hawaiian and Other Pacific Islander, or other nonspecified). Compared with participants without a history of correctional control, those with a personal history of incarceration were at greater odds of having an emergency department visit that did not result in hospitalization in models adjusted for age, sex, and race and ethnicity (odds ratio, 2.87; 95% CI, 1.47-5.75).

**Conclusions and Relevance:**

This cross-sectional study suggests that primary care clinicians should screen for correctional control as a prevalent social determinant of health.

## Key Points

**Question:**

What proportion of patients in an urban primary care clinic have an individual or family history of correctional control, and how is this history associated with patients’ perceived and objective health outcomes?

**Findings:**

In this cross-sectional, mixed-methods study, 39% of 200 primary care patients surveyed had a history of incarceration, and 46% reported having a family member with a history of incarceration. Participants with a personal history of incarceration were significantly more likely to have an emergency department visit that did not result in hospitalization compared with those without a history of correctional control.

**Meaning:**

This study suggests that primary care clinicians should screen patients for correctional control as a prevalent social determinant of health.

## Teaser

This cross-sectional study uses patient surveys and medical record review to quantify the proportion of patients in an urban primary care clinic with an individual or family history of incarceration, probation, and/or parole and to evaluate how correctional control is associated with health outcomes.

## Introduction

The US incarcerates people at higher rates than any other country in the world.^1^ Currently, 1 in every 37 people in the US are under some form of correctional control, including prison, jail, probation, and parole.^2,3^ Although Rhode Island is known for having one of the lowest incarceration rates in the country, it has the second-highest probation rate.^4^ Community supervision, composed of either probation or parole, is defined by long supervision terms, strict requirements, and far-reaching surveillance.^4,5^ Widespread correctional control disproportionately affects racial and ethnic minority groups, particularly Black communities, both nationwide and at the state level.^4,5,6,7^ Black people make up only 6% of Rhode Island’s population but 30% of those in prisons and jails.^2^

Research has shown that experiences of incarceration and community supervision are associated with worse health outcomes for incarcerated individuals, their families, and their communities both during and after incarceration.^6,7,8,9,10,11^ Compared with the general population, formerly incarcerated individuals are more likely to experience chronic health conditions and substance use disorders owing to challenges with social integration, reintegration, and stress.^12,13^ Each year served in prison is associated with a 2-year decrease in life expectancy.^14^ Furthermore, research has continued to demonstrate the association of family history of incarceration with health, specifically including negative health outcomes for children, parents, and partners of incarcerated individuals.^15,16,17,18,19^

The diminished health outcomes of those with involvement in correctional control cannot be disentangled from the barriers to basic rights and well-being that persist even after release from correctional control.^20,21^ Discrimination in housing and employment, difficulty accessing transportation and social services, and fragmented transitional health care are all aspects of structural violence that shape health inequalities for formerly incarcerated individuals.^5,6,22^ Establishing primary care and managing chronic illness are often lower priorities for formerly incarcerated people than finding housing, employment, and food.^23^ However, engaging individuals in primary care soon after release from prison has been associated with benefits, including increasing use of primary care and decreasing use of emergency department (ED) care.^24,25^

Despite understanding the benefits associated with engaging recently released individuals in primary care, additional research is needed to understand the unique ways in which primary care patients’ health outcomes are associated with both personal and family experiences of correctional control. Limited research has explored whether individuals and families perceive that there is an association between incarceration and their health. Therefore, we chose to use a mixed-methods approach to address this research gap. This cross-sectional study aimed to (1) quantify the proportion of patients in a primary care clinic who had an individual or family history of incarceration, probation, and/or parole and (2) evaluate how correctional control was associated with patient health based on subjective and objective health outcomes. We hypothesized that a substantial portion of patients in a primary care setting would have an individual and/or family history of correctional control and that this history would be negatively associated with subjective and objective health outcomes.

## Methods

### Recruitment and Data Collection

The Rhode Island Hospital Center for Primary Care (CPC) is a multidisciplinary, urban primary care clinic based in South Providence that serves approximately 9000 patients per year. The CPC is the academic primary care clinic for Brown University’s internal medicine residency program and serves a predominantly underinsured patient population. Most CPC patients live in Providence, Rhode Island’s capital city of 180 000 people.^26^ Compared with the national average, Rhode Island has a low incarceration rate of 361 per 100 000, but it has among the highest probation rates, with 1 in 35 adult residents on probation supervision.^4^ From July 9, 2019, to January 10, 2020, CPC patients, regardless of their length of clinic use, were asked if they wanted to participate in a survey that was administered in the time between being seated in their private examination room and seeing their health care professional. The eligibility criteria for this cross-sectional study were being 18 years of age or older and being able to complete a survey in English. Patients were given the option to skip or refuse questions or opt out of the study completely. This study was approved by the Lifespan institutional review board and followed the Strengthening the Reporting of Observational Studies in Epidemiology (STROBE) reporting guideline for cross-sectional studies and the Standards for Reporting Qualitative Research (SRQR) reporting guideline for qualitative studies.^27,28^

Research assistants (O.M., M.C., and B.D.) provided a description of the survey and obtained written consent from the participants. Once consent was obtained, research assistants administered a survey that asked 20 questions about perceived health and personal or familial experiences of incarceration, probation, and parole. To assess perceived health, all participants were asked, “In general, how would you rate your overall health?” and responded on a 5-point Likert scale (where 1 indicated excellent health and 5 indicated poor health). Participants who indicated that they had personally experienced incarceration, probation, and/or parole were then asked if they felt that this experience impacted their health on a 3-point scale: “It improved my health,” “It did not impact my health,” and “It worsened my health.” Participants were given the opportunity to answer any of 3 open-ended questions that were applicable to their circumstances: “How did your family member being incarcerated affect your health?,” “How did being in prison or jail affect your health?,” and/or “How has being on probation or parole affected your health?” Given the time-limited nature and setting of the interviews, qualitative responses to the open-ended questions were written down by research assistants rather than audio-recorded.

Research assistants abstracted several demographic characteristics and key health indicators from the electronic medical record. Demographic data included age, sex, and race and ethnicity. Race and ethnicity data were captured directly from the electronic medical record and are therefore a combination of patient self-report and clinical staff assumptions. Key health indicators included the number of chronic conditions and the number of medications. The number of chronic illnesses of each patient were counted using *International Statistical Classification of Diseases and Related Health Problems, Tenth Revision* codes. Chronic conditions were defined using 21 categories set by the Centers for Medicare & Medicaid Services.^29^ All participants were given a random study identification number that was used to link their survey responses with a retrospective medical record review to supplement self-reported health outcomes.

To account for substantial variation in the number of chronic illnesses experienced by participants, this measure was collapsed such that participants were classified as having 0, 1 to 5, or 6 or more chronic conditions. To understand health care use, research assistants abstracted the number of hospital admissions and ED visits that did not result in hospital admission, which was used as an index of overreliance on the ED for health care, in the 365 days prior to survey administration.^30^ These measures were then collapsed into binary variables, with categories being 0 ED visits or admissions and 1 or more ED visits or admissions. To capture primary care engagement, research assistants also reviewed medical records to see if patients had received select preventive health screenings, including those for breast, colon, and cervical cancer; the appropriateness and eligibility of screenings were based on criteria set by the US Preventive Services Task Force.^31,32,33^ After each value was abstracted, a dichotomous measure was created that captured whether participants had received all applicable preventive screenings or not. Although this measure does not include all screenings recommended by the US Preventive Services Task Force, breast, colon, and cervical cancer screenings were chosen as representative measures because of both their clinical importance and their ease of verifiability in medical record review.

### Statistical Analysis

Two separate quantitative analyses were completed to understand how correctional control was associated with patient health at the CPC. Demographic and health characteristics were compared between participants with and participants without personal or familial histories of incarceration. For all comparisons, descriptive statistics were generated for demographic and health-related factors, such as age, sex, race and ethnicity, self-reported health status, blood pressure, and health care use. The association of age with correctional control status was evaluated using independent-sample *t* tests. For all other factors, the Fisher exact test was performed to measure the potential association of these categorical correlates with personal or familial experiences of incarceration.

In addition to these analyses, exploratory multivariable analyses were performed to assess whether having a personal history of incarceration or having a family member with a history of incarceration was associated with the risk of not having up-to-date selected, preventive health screenings or having 1 or more ED visits that did not result in hospitalization. To complete these exploratory analyses, 3 nested logistic models were created to assess whether the exposures of personal or family history of incarceration were each associated with either not having up-to-date preventive screenings or having 1 or more ED visits that did not result in hospitalization. For each of these exposure and outcome pairs, 3 nested logistic regression models were estimated. Odds ratios and corresponding 95% CIs are reported. Model 1 was adjusted for age. Model 2 adjusted for age, sex, and race and ethnicity. Model 3 adjusted for age, sex, race and ethnicity, and number of chronic conditions. Chronic conditions are likely on the causal pathway between correctional control and health care use and therefore are not the primary aim of analysis.^23^ For each model, logistic regression was used. A final model, model 4, was created to evaluate whether having a family member with a history of incarceration confounded the association between personal history of incarceration and the study’s 2 outcomes. However, personal history of incarceration was not included as a covariate when examining the association between having a family member with a history of incarceration and each outcome because personal history of incarceration may be a mediator of the association. One participant was removed from the full analytic sample because of missing ethnicity data. An additional 3 participants were removed in analyses pertaining to having a family member with a history of incarceration because they skipped that question. For all tests, 2-sided *P* values were used and were considered statistically significant at *P* < .05. Analysis was performed using RStudio software, version 1.2.5001 (RStudio).

Qualitative analysis of the participants’ responses to open-ended questions was conducted by 2 of the authors independently (M.C. and B.D.). With the use of an immersion/crystallization approach, the responses to 3 open-ended questions were continuously reviewed by each author until themes were derived.^34^ Themes were further developed based on a joint review of responses and refined in an iterative process using consensus. A comment could contain more than 1 theme.

## Results

A total of 200 participants were interviewed; 1 participant was removed from the final analytic sample owing to missing ethnicity data. Participants’ characteristics are summarized in Table 1. The study population was predominantly male (113 of 199 [56.8%]) and non-Hispanic (157 of 199 [78.9%]), with a mean (SD) age of 51.2 (14.0) years. Eighty-six of 199 individuals (43.2%) identified as female. A total of 62 of 199 (31.2%) identified as non-Hispanic Black, 93 of 199 participants (46.7%) identified as non-Hispanic White, and 44 of 199 (22.1%) identified as belonging to another race (American Indian and Alaska Native, Asian, Native Hawaiian and Other Pacific Islander, or other nonspecified). Overall, 31 of 199 participants (15.6%) reported excellent or very good health, 144 of 199 (72.4%) reported good or fair health, and 22 of 199 (11.1%) reported poor health. For patients with a history of incarceration (n = 78), 10 (12.8%) reported excellent or very good health, 59 (75.6%) reported good or fair health, and 8 (10.3%) reported poor health. For patients without a history of incarceration (n = 121), 21 (17.4%) reported excellent or very good health, 85 (70.2%) reported good or fair health, and 14 (11.6%) reported poor health. Of the 199 participants, 78 (39.2%) reported a personal history of incarceration, 32 (16.1%) reported being currently supervised by probation or parole, and 92 (46.2%) reported having a family member with a history of incarceration.

**Table 1.** Demographic and Health Characteristics of Patients Receiving Primary Care at the Center for Primary Care in 2019

Characteristic	No. (%) (N = 199)
Sex	
Male	113 (56.8)
Female	86 (43.2)
Age, mean (SD), y	51.2 (14.0)
Race	
Black or African American	62 (31.2)
White	93 (46.7)
Other^a^	44 (22.1)
Ethnicity	
Hispanic	42 (21.1)
Non-Hispanic	157 (78.9)
Self-reported health status	
Excellent or very good	31 (15.6)
Good or fair	144 (72.4)
Poor	22 (11.1)
Do not know or refuse	2 (1.0)
All recommended screenings up to date^b^	
Yes	151 (75.9)
No	48 (24.1)
Blood pressure	
Normal	57 (28.6)
Elevated	25 (12.6)
Hypertensive	117 (58.8)
Pulse, mean (SD), beats/min	81.4 (14.6)
No. of chronic illnesses	
0	21 (10.6)
1-5	81 (40.7)
≥6	97 (48.7)
No. of medications being taken daily, mean (SD)	8.5 (5.6)
No. of visits to primary care clinician in the last year	
0	45 (22.6)
1-4	129 (64.8)
≥5	25 (12.6)
No. of emergency department visits in the last year^c^	
0	95 (47.7)
≥1	104 (52.3)
No. of hospital admissions in the last year	
0	143 (71.9)
≥1	56 (28.1)
Any family members been incarcerated?	
No	104 (52.3)
Yes	92 (46.2)
Do not know or refuse	3 (1.5)
Has personal health been impacted by family members’ incarceration?	
No.	92
No	52 (56.5)
Yes	40 (43.5)
Personal experience of incarceration	
No	121 (60.8)
Yes	78 (39.2)
How did prison impact your health?	
No.	78
It improved my health	5 (6.4)
It did not impact my health	23 (29.5)
It worsened my health	50 (64.1)
Are you currently being supervised on probation or parole?	
No	165 (82.9)
Yes	32 (16.1)
Do not know or refuse	2 (1.0)
How has probation or parole affected your health?	
No.	32
It improved my health	2 (6.3)
It did not impact my health	21 (65.6)
It worsened my health	8 (25.0)
Do not know or refuse	1 (3.1)

^a^ Included American Indian and Alaska Native, Asian, Native Hawaiian and Other Pacific Islander, or other nonspecified.

^b^ Recommended screenings were defined by the US Preventive Services Task Force guidelines for breast cancer, colon cancer, and cervical cancer screenings.

^c^ Defined as emergency department visits that did not result in hospital admission.

Qualitative results can be found in the Figure. Of the 78 participants who reported experiencing incarceration, 50 (64.1%) reported that incarceration worsened their health. This outcome was also evident in the qualitative data, which found that several negative health outcomes were reported, including depression and sadness (19 [24.4%]), lack of adequate medical care (18 [23.1%]), stress (17 [21.8%]), anxiety (14 [17.9%]), and unhealthy environmental conditions associated with morbidity (4 [5.1%]), such as asthma exacerbation and skin and soft tissue infection. Five participants (6.4%) identified incarceration as improving their health. Among these participants, access emerged as the major theme, including lack of access to substances, improved access to chronic condition management, and improved access to food. Twenty-three participants (29.5%) stated that incarceration did not affect their health.

**Figure.**
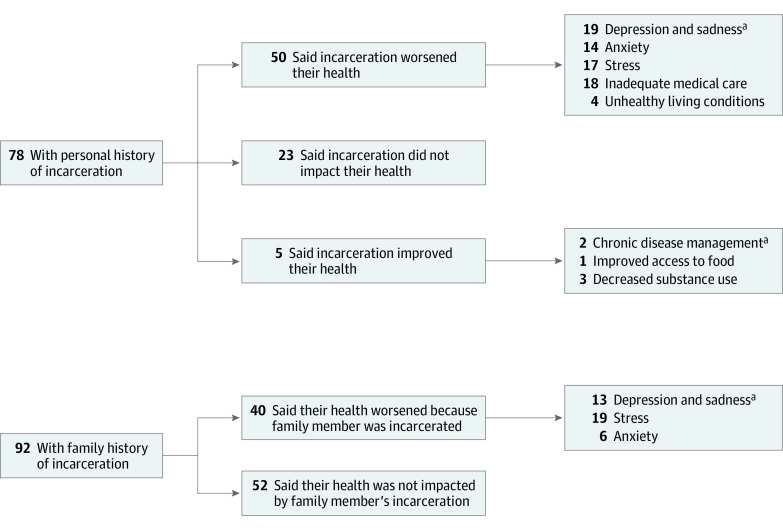
Results From Qualitative Analysis of the Association Between Personal or Family History of Correctional Control and Health ^a^A comment could contain more than 1 theme, so numbers sum to more than the total number.

Of the 92 participants with a family member who had been incarcerated, 40 (43.5%) indicated that they believed their own health was affected because of the family member’s incarceration. These respondents primarily identified the experience as having a negative association with their own mental health, citing significant stress (19 [20.7%]), depression and sadness (13 [14.1%]), and anxiety (6 [6.5%]).

Differences in demographic and health characteristics by personal experience of incarceration can be found in Table 2. Those who had personal experiences of incarceration were more likely than those without such experiences to be male (60 of 78 [76.9%] vs 53 of 121 [43.8%]; *P* < .001), have a family with a history of incarceration (49 of 78 [62.8%] vs 43 of 121 [35.5%]; *P* < .001), and have had 1 or more ED visits that did not result in hospitalization (50 of 78 [64.1%] vs 54 of 121 [44.6%]; *P* = .009). There were no statistically significant differences in other demographic characteristics, self-reported health, blood pressure, or number of chronic illnesses.

**Table 2.** Demographic and Health Characteristics of Patients Receiving Care at the Center for Primary Care in 2019 Stratified by Personal Experience of Incarceration (N = 199)

Characteristic	Personal experience of incarceration, No. (%)		*P* value
	No (n = 121)	Yes (n = 78)	
Sex			
Male	53 (43.8)	60 (76.9)	<.001
Female	68 (56.2)	18 (23.1)	
Age, mean (SD), y	52.6 (13.8)	49.2 (14.0)	.08
Race			
Black or African American	37 (30.6)	25 (32.1)	
White	55 (45.5)	38 (48.7)	.76
Other^a^	29 (24.0)	15 (19.2)	
Ethnicity			
Hispanic	31 (25.6)	11 (14.1)	.07
Non-Hispanic	90 (74.4)	67 (85.9)	
Self-reported health status			
Excellent or very good	21 (17.4)	10 (12.8)	.78
Good or fair	85 (70.2)	59 (75.6)	
Poor	14 (11.6)	8 (10.3)	
Do not know or refuse	1 (0.8)	1 (1.3)	
Family member with history of incarceration			
No	76 (62.8)	28 (35.9)	<.001
Yes	43 (35.5)	49 (62.8)	
Do not know or refuse	2 (1.7)	1 (1.3)	
All screenings up to date^b^			
No	30 (24.8)	18 (23.1)	.87
Yes	91 (75.2)	60 (76.9)	
Blood pressure			
Normal	28 (23.1)	29 (37.2)	.09
Elevated	15 (12.4)	10 (12.8)	
Hypertensive	78 (64.5)	39 (50.0)	
Pulse, mean (SD), beats/min	81.8 (14.2)	81.1 (15.0)	.73
No. of chronic illnesses			
0	15 (12.4)	6 (7.7)	.21
1-5	53 (43.8)	28 (35.9)	
≥6	53 (43.8)	44 (56.4)	
No. of medications being taken daily, mean (SD)	8.9 (6.0)	7.7 (4.7)	
No. of visits to primary care clinician in the last year			
0	24 (19.8)	21 (26.9)	.41
1-4	82 (67.8)	47 (60.3)	
≥5	15 (12.4)	10 (12.8)	
No. of emergency department visits in the last year^c^			
0	67 (55.4)	28 (35.9)	.009
≥1	54 (44.6)	50 (64.1)	
No. of hospital admissions in the last year			
0	92 (76.0)	51 (65.4)	.11
≥1	29 (24.0)	27 (34.6)	

^a^ Included American Indian and Alaska Native, Asian, Native Hawaiian and Other Pacific Islander, or other nonspecified.

^b^ Recommended screenings were defined by the US Preventive Services Task Force guidelines for breast cancer, colon cancer, and cervical cancer screenings.

^c^ Defined as emergency department visits that did not result in hospital admission.

Differences in demographic and health characteristics by familial experience of incarceration can be found in Table 3. Unlike those with a personal history of incarceration, those with a familial experience of incarceration compared with those without were less likely to be male (44 of 92 [47.8%] vs 66 of 104 [63.5%]; *P* = .03), have personal experience of incarceration (49 of 92 [53.3%] vs 28 of 104 [26.9%]; *P* < .001), and have 6 or more chronic illnesses (55 of 92 [59.8%] vs 42 of 104 [40.4%]; *P* = .02) but less likely to have 1 to 5 illnesses (28 of 92 [30.4%] vs 50 of 104 [48.1%]; *P* = .02).

**Table 3.** Demographic and Health Characteristics of Patients Receiving Care at the Center for Primary Care in 2019 Stratified by Whether the Patient Has a Family Member With a History of Incarceration^a^

Characteristic	Family member with history of incarceration, No. (%)		*P* value
	No (n = 104)	Yes (n = 92)	
Sex			
Male	66 (63.5)	44 (47.8)	.03
Female	38 (36.5)	48 (52.2)	
Age, mean (SD), y	52.8 (13.9)	49.2 (14.1)	.08
Race			
Black or African American	26 (25.0)	36 (39.1)	.08
White	55 (52.9)	36 (39.1)	
Other^b^	23 (22.1)	20 (21.7)	
Ethnicity			
Hispanic	24 (23.1)	18 (19.6)	.60
Non-Hispanic	80 (76.9)	74 (80.4)	
Self-reported health status			
Excellent or very good	19 (18.3)	12 (13.0)	.28
Good or fair	76 (73.1)	66 (71.7)	
Poor	9 (8.7)	12 (13.0)	
Do not know or refuse	0	2 (2.2)	
All screenings up to date^c^			
No	25 (24.0)	23 (25.0)	>.99
Yes	79 (76.0)	69 (75.0)	
Personal history of incarceration			
No	76 (73.1)	43 (46.7)	<.001
Yes	28 (26.9)	49 (53.3)	
Blood pressure			
Normal	27 (26.0)	30 (32.6)	.43
Elevated	12 (11.5)	13 (14.1)	
Hypertensive	65 (62.5)	49 (53.3)	
Pulse, mean (SD), beats/min	82.5 (14.4)	81.0 (14.9)	.49
No. of chronic illnesses			
0	12 (11.5)	9 (9.8)	.02
1-5	50 (48.1)	28 (30.4)	
≥6	42 (40.4)	55 (59.8)	
No. of medications taken daily, mean (SD)	8.4 (6.1)	8.4 (4.7)	.97
No. of visits to primary care clinician in the last year			
0	22 (21.2)	21 (22.8)	.81
1-4	70 (67.3)	58 (63.0)	
≥5	12 (11.5)	13 (14.1)	
No. of emergency department visits in the last year^d^			
0	52 (50.0)	41 (44.6)	.48
≥1	52 (50.0)	51 (55.4)	
No. of hospital admissions in the last year			
0	73 (70.2)	68 (73.9)	.62
≥1	31 (29.8)	24 (26.1)	

^a^ Three participants were removed from the sample resulting from the fact that they did not answer the question regarding whether they had a family member with a history of incarceration. As a result, there are only 196 participants in this sample.

^b^ Included American Indian and Alaska Native, Asian, Native Hawaiian and Other Pacific Islander, or other nonspecified.

^c^ Recommended screenings were defined by the US Preventive Services Task Force guidelines for breast cancer, colon cancer, and cervical cancer screenings.

^d^ Defined as emergency department visits that did not result in hospital admission.

Results from the series of exploratory multivariable analyses are found in Table 4. In analyses adjusted for age, sex, and race and ethnicity, those with a personal history of incarceration were twice as likely to have an ED visit that did not result in hospitalization compared with those without a correctional history (odds ratio, 2.87; 95% CI, 1.47-5.75). Additional adjustment for the number of chronic conditions yielded results that were no longer statistically significant. Personal history of incarceration did not significantly increase the odds of not having up-to-date health maintenance screenings. Having a family member with a history of incarceration did not result in a statistically significant risk of increased ED use or odds of not having all health maintenance screenings up to date. Finally, the results for having a personal history of incarceration and having all screenings up to date (odds ratio, 0.95; 95% CI, 0.01-1.02) or having a personal history of incarceration and having 1 or more ED visit that did not result in hospitalization (odds ratio, 0.99; 95% CI, 95.2-1.02) was materially unchanged after adjusting for having a family member with a history of incarceration.

**Table 4.** Results From Multivariable Analyses of the Risk of Not Having Up-to-Date Screenings and Having 1 or More ED Visits Not Resulting in Hospitalization, by History of Incarceration^a^

Characteristic	Odds ratio (95% CI)	
	Personal history of incarceration (n = 78)	Having a family member with a history of incarceration (n = 92)
All screenings not up to date, No. (%)		
Yes	60 (76.9)	69 (75.0)
No	18 (23.1)	23 (25.0)
Model 1	1.00 (0.50-1.96)	1.18 (0.60-2.31)
Model 2	1.51 (0.70-3.25)	1.08 (0.54-2.18)
ED visits not resulting in hospitalization, No. (%)		
0	28 (35.9)	41 (44.6)
≥1	50 (64.1)	51 (55.4)
Model 1	2.11 (1.17-3.84)	1.17 (0.96-1.00)
Model 2	2.87 (1.47-5.75)	1.18 (0.64-2.17)

Abbreviation: ED, emergency department.

^a^ Model 1 is adjusted for the exposure of interest and age, and model 2 is adjusted for the exposure of interest, age, sex, and race and ethnicity.

## Discussion

In this mixed-methods, cross-sectional study, nearly 40% of participants surveyed had a history of incarceration, and 16.1% were on probation or parole at the time of the study. This finding speaks to the high rates of incarceration in the US and, in particular, the high rates of incarceration of racial and ethnic minority groups, specifically Black individuals, in the US.^5,9^ Although Black persons comprise 16% of the Providence population, they were overrepresented among respondents with a personal history of incarceration (62 of 199 [31.2%]), underscoring how racial and ethnic minorities are disproportionately represented in the criminal legal system.^35,36^ That 46.2% of participants had a family member who has been incarcerated underscores the far-reaching effects of mass incarceration. This finding is not unique to this primary care facility. A study conducted in multiple community clinics in the Bronx, New York, found that more than half of the families seen in the clinics had experienced arrest and incarceration.^37^ Our findings lend additional credence to the notion that high rates of correctional control are prevalent among urban primary care populations and highlight the importance of routine screening for a history of correctional control among patients engaging in primary care.

Participants with a history of incarceration had twice the odds of having an ED visit that did not result in hospitalization compared with those without a history of incarceration. Although there were no statistically significant differences in other key health indicators in participants’ medical records between those with and those without personal histories of incarceration, most patients with a history of incarceration perceived their time in prison as harmful to their health. In concordance with previous literature, these findings show that patients with involvement in correctional control have higher health care use, which could be explained by the challenges of navigating the health care system while dealing with the negative health outcomes associated with incarceration.^38,39^

Although it has become more commonplace for primary care clinicians to screen for social determinants of health, it is essential that these screenings also include involvement in correctional control, both as a determinant in itself and as an exacerbator of all downstream health barriers. Clinicians may be wary of screening for involvement in correctional control, citing apprehension of asking questions that they do not have the training or resources to address.^40^ However, this scenario does not need to be the case. Knowledge of a patient’s involvement in correctional control allows clinicians to anticipate health sequelae associated with the structural violence of incarceration, including overdose, suicide, and increased reliance on the ED for health care, as borne out in our study.^5,41^ In addition, screening for any social determinant of health is best done in a team-based care setting in which clinicians collaborate with community health care workers and social workers.^42^

Knowledge that correctional control is negatively associated with patient health must also translate to advocacy. Primary care clinicians are uniquely positioned to advocate for their patients with involvement in correctional control.^43,44^ Successful models have demonstrated the utility of medical-legal partnerships, as well as the use of form letters and other clinician advocacy tools to prevent incarceration and waive court debt.^45^

### Limitations

There were several limitations to this study. First, the small sample size and reliance on data from 1 study site limit generalizability of the results and thus warrant a larger study to further assess the associations between incarceration and health. In addition, the small sample size may have resulted in insufficient power to reject the null hypothesis for the univariable and multivariable models.^46^ However, point estimates, particularly when examining personal history of incarceration, were not null; rather, they were all positive but yielded wide 95% CIs. This finding suggests that our models were underpowered to detect significance. Furthermore, in assessing the association between family history of incarceration and both ED use and having up-to-date screenings, the wide 95% CIs suggest that our precision was limited, which may have affected our ability to find subtle signals. In addition, this was a cross-sectional study and therefore was limited in its ability to explore the temporal association between incarceration and health outcomes. In particular, ED visit data were extracted for the 12 months prior to the date of survey administration, complicating our ability to know whether ED visits had occurred before or after a participant’s involvement in correctional control. Future studies could use alternative study designs to comment on these temporal associations.

Second, there was a possibility of sampling bias given that patients with incarceration experience may have been more willing to participate in the study. Furthermore, participants in our study may not be fully reflective of the general clinic patient population. Although many patients who were screened did choose to participate in the study, the results of this study would be strengthened if a larger group of patients were sampled. Sampling bias may also have been introduced through the complete case analysis, and although only 4 participants were removed, we cannot assume that the missing data were random. In addition, we were able to conduct this survey only among English-speaking participants. Future studies examining experiences of correctional control in the primary care setting should include Spanish-speaking and other non–English-speaking patients, especially as non–English-speaking people make up an increasing subgroup in the US prison system.^47^

Third, although every attempt was made to record qualitative responses verbatim, we cannot ensure that this process always occurred because responses were not audio-recorded, which limited our qualitative analysis to only the crystallized themes that emerged from the data. Further qualitative research is needed to understand more robustly how experiences of correctional control shape health and health care access from the patient perspective. In addition, our survey instrument was not previously validated. Fourth, patients were recruited from an urban primary care facility that serves patients who are more likely to be underinsured and have more chronic medical conditions than patients in other health care settings; this factor may have restricted the extent to which meaningful differences could be detected in subjective and objective health outcomes between those with and those without a history of correctional control.

## Conclusions

This cross-sectional study found that a substantial proportion of individuals presenting for primary care in an urban setting had a personal history of incarceration and indicated that the experience of incarceration had worsened their health. In addition, patients with involvement with correctional control were significantly more likely to present to the ED for health care. Given the extensive prior literature on the negative association of incarceration with health outcomes, this study’s finding of the widespread prevalence of correctional control in the primary care setting positions primary care clinicians as first responders to the epidemic of mass incarceration and further supports recent calls to expand the traditional role of the primary care clinician.
